# Indocyanine green intravenous administration can more accurately identify the intersegmental plane than the inflation-deflation method in lung segmentectomy

**DOI:** 10.1371/journal.pone.0328362

**Published:** 2025-08-04

**Authors:** Takahiro Ochi, Yuichi Sakairi, Yuki Sata, Takahide Toyoda, Terunaga Inage, Kazuhisa Tanaka, Hajime Tamura, Masako Chiyo, Yukiko Matsui, Yuki Shiko, Masayuki Ota, Jun-ichiro Ikeda, Ichiro Yoshino, Hidemi Suzuki

**Affiliations:** 1 Department of General Thoracic Surgery, Chiba University Graduate School of Medicine, Chiba, Japan; 2 Division of Thoracic Surgery, Chiba Cancer Center, Chiba, Japan; 3 Clinical Research Center, Chiba University Hospital, Chiba, Japan; 4 Department of Diagnostic Pathology, Chiba University Graduate School of Medicine, Chiba, Japan; 5 Department of Thoracic Surgery, International University Health and Welfare School of Medicine, Narita, Japan; Taichung Veterans General Hospital, TAIWAN

## Abstract

**Background:**

Indocyanine green (ICG) intravenous administration (ICG-iv) has been described for detecting the intersegmental plane in lung segmentectomy. However, errors with preoperative planning and accuracy comparisons with alternative methodologies have not been fully validated.

**Methods:**

This single-center retrospective study identified 138 patients with 140 lesions who underwent segmentectomy using the inflation-deflation (I-D) method or ICG-iv method. The planned margin was calculated using three-dimensional imaging, and the surgical margin was measured for the resected specimen. We evaluated the surgical and planned margin ratio (S/P ratio) and log S/P ratio. Accuracy was also tested using the Root Mean Squared Logarithmic Error (RMSLE): the smaller the RMSLE, the more accurate.

**Results:**

The study enrolled 86 patients with 88 lesions in the I-D group and 52 patients with lesions in the ICG-iv group. All lesions were completely resected. The ICG-iv group underwent significantly more complex segmentectomies compared to the I-D group (*P* < 0.001). The median S/P ratio was 0.886 (I-D) and 0.912 (ICG-iv). The mean log S/P ratio was −0.061 (I-D) and −0.013 (ICG-iv). The RMSLE values were 0.258 (I-D) and 0.229 (ICG-iv). In the ICG-iv group, eight patients with lesions (15.3%) had poor staining for intersegmental identification. Notably, the poor staining subgroup included a higher proportion of patients with obstructive pulmonary disease (4/8: 50.0%) compared to the good staining group (6/44: 13.6%) (**P* *= 0.035).

**Conclusions:**

The ICG-iv method demonstrated superior accuracy in identifying the intersegmental plane compared to I-D method; however, concerns persist regarding suboptimal staining in patients with obstructive pulmonary disease.

## Introduction

A randomized trial comparing segmentectomy with lobectomy in small peripheral non-small cell lung cancers showed the superiority of segmentectomy [1]. In addition, sublobar resection, including wedge resection and segmentectomy, for similar lesions was shown to have comparable results to lobectomy [2]. However, the risk of local recurrence after segmentectomy has also remained. Better visualization of the intersegmental plane, a three-dimensional boundary between segments, during surgery is thought to have the potential to contribute to a reduction in local recurrence.

At our institution, the inflation-deflation (I-D) method, a technique to inflate the collapsed lung and confirm the boundary between the collapsed and inflated areas, has been actively adopted to identify the segment to be resected. In some cases, however, intersegmental visualization was suboptimal due to the condition of the lung, and the time loss associated with inflating and subsequently deflating the lung again was also a notable issue. To address these issues, indocyanine green (ICG) intravenous administration (ICG-iv) method was introduced since September 2021. The usefulness of ICG-iv method to detect referred pulmonary segments by confirming the boundary between unstained and stained areas by ICG after vascular transection, has been reported in retrospective analyses [3–11]. However, errors with preoperative planning and accuracy comparisons with alternative approaches, including the I-D method, have not been fully validated. Thus, we aimed to verify and compare the accuracy of the I-D and ICG-iv methods.

## Materials and methods

### Study design

This retrospective study was approved by the Ethics Committee of Chiba University Graduate School of Medicine (No. M10556, 1/23/2023) and complied with the tenets of the Declaration of Helsinki. The requirement for informed consent from each participant was waived. A total of 138 patients with 140 lesions underwent segmentectomy using either the I-D or ICG-iv methods from January 2020 to December 2022. The data was collected during the period from March 22nd to May 16th, 2023. During data collection, the author had access to information that could identify individual participants, but after the collection, the data was anonymized.

The patients enrolled in this study included those who underwent segmentectomy as a curative intended and compromised adaptation. In patients who underwent segmentectomy as a curative adaptation, a planned margin of at least 20 mm or larger than the tumor diameter was assumed preoperatively. Even in patients who underwent segmentectomy as a compromised adaptation, only cases with a planned margin of at least 5 mm were included. This criterion was determined based on the assumption that when a tumor is located near or just on the intersegmental border, a more extensive resection is required and determined surgical margin during the operation. Thus, the margin cannot measure preoperative image-based measurement, and such cases did not match our research focus and were excluded from this analysis. Simple segmentectomy specifies the superior segments, basal segments, left upper division, and left lingular segments as the targets, whereas complex segmentectomy targets the rest of the segments. Postoperative prolonged air leakage in this report was defined as grade three or higher according to the Common Terminology Criteria for Adverse Events version 5.0.

### Operative methods

**Fig 1:**
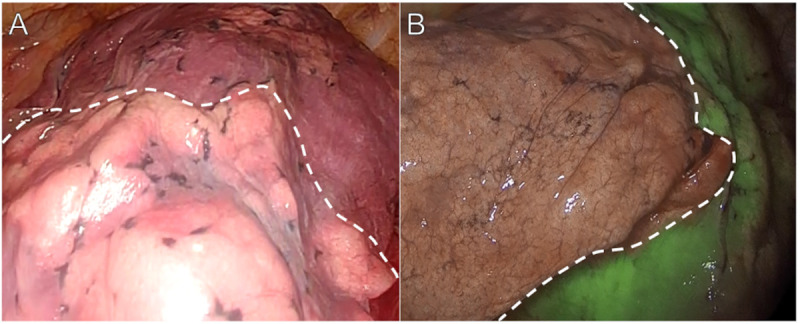
Intraoperative findings of the intersegmental plane description using the I-D method and ICG-iv method. (A) I-D method. (B) ICG-iv method.

In the I-D method, after the target area bronchus is dissected, the other pulmonary regions are inflated with air to identify the intersegmental plane (Fig 1A). In the ICG-iv method, the concentration of the ICG solution was adjusted to 2.5 mg/mL with distilled water, as in previous reports [3–5]. After resection of the pulmonary arteries and veins of the subject segment, the ICG solution was administered intravenously and the continuous infusion flow rate was temporarily accelerated: the dose of the ICG solution did not exceed 0.5 mg/kg. A near-infrared thoracoscope was used to detect ICG fluorescence and to determine the intersegmental plane for pulmonary segmentectomy (Fig 1B). However, there were some patients with lesions whose intersegmental planes were poorly stained using the ICG-iv method in this study. Poor staining indicates that the intended intersegmental plane is not appropriately visualized, such as when the delineated line by ICG lacks linearity (S1 Fig). In case of poor staining, additional measures, such as the I-D method and/or identification of the intersegmental veins to the periphery, were used to determine the intersegmental plane. To validate the factors involved in the ability of ICG to show the intersegmental plane, patient and surgical characteristics were compared for the good and poor staining groups.

Before lung resection, tumor localization was confirmed by visual inspection and/or palpation. In cases where the tumor was located slightly deeper from the pleural surface, it was confirmed by palpation after resection. If necessary, the specimen was incised to allow visual inspection.

### ICG-iv, Indocyanine green intravenous administration; I-D, Inflation-deflation

#### Assessment methods.

**Fig 2:**
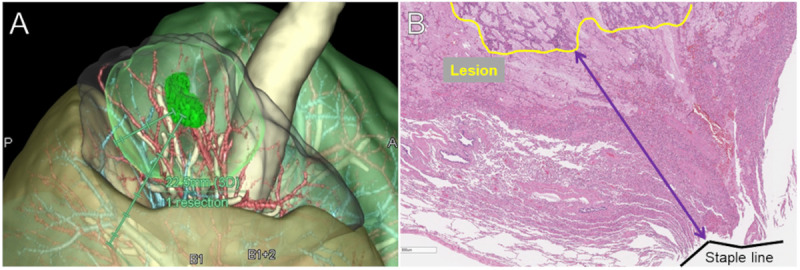
Calculation of margins. (A) The planned margins were calculated using Ziostation (Ziosoft, Tokyo, Japan) based on CT data. (B) The surgical margins were measured as the distance between the tumor rim and the resection edge in the resected specimen.

The preoperative planning margins (planned margin) were calculated using Ziostation (Ziosoft, Tokyo, Japan) based on CT data (Fig 2A). The distance between the tumor rim and the resection edge was calculated (surgical margin) in the resected specimen; naturally, it was also pathologically validated to see if the complete resection could be archived (Fig 2B). For the pathological evaluation, formalin solution was injected through the resected bronchus and inflated for accurate margin measurement. For all patients enrolled in the study, an automatic suture device was used to dissect the intersegmental plane, and thus, an additional 5 mm was added uniformly as a staple thickness to measure the margin. Spread through air spaces (STAS) was also confirmed, as it has been reported to be a poor prognostic factor in lung cancer [12]. Among STAS lesions, the distance of STAS from the primary tumor was measured as the distance between the tumor rim and the distal end of the STAS. We also compared this distance with the surgical margin to evaluate the suitability of the surgical procedure. All pathological evaluations were performed by expert pathologists.

### Statistical analysis

Based on the surgical/planned margin ratio (S/P ratio), the logarithmic transformation of the S/P ratio (log S/P ratio), used to uphold the assumptions of a linear mixed model (including normal distribution of residuals), was performed [13,14]. Log S/P ratio is a key metric for evaluating the precision of surgical planning and execution. We also selected the Root Mean Squared Logarithmic Error (RMSLE) over other potential error metrics because of its specific advantages. RMSLE quantifies the discrepancy between predicted and actual values based on their relative differences, making it particularly useful for skewed distributions or in cases where proportional errors are more informative than absolute ones. A smaller RMSLE indicates higher predictive accuracy, which is especially important in surgical margin evaluation, where large absolute errors may not necessarily reflect clinically significant deviations, while proportional errors can provide better insights into surgical precision.


$$RMSLE=\sqrt{\frac{1}{N}\sum_{i=1}^{N}{\left(\log\left(1+{\hat{y}}_{i}\right)-\log\left(1+y_{i}\right)\right)}^{2}}$$

${\hat{y}}_{i}$=Predicted value,$,\;y_{i}$=Actual value

Propensity scores were estimated via logistic regression modeling, incorporating the following clinical covariates: sex, smoking history, restrictive pulmonary disease, obstructive pulmonary disease, lower lobe segmentectomy, complex segmentectomy, and consolidation/tumor ratio. One-to-one propensity score matching was subsequently conducted using the nearest-neighbor algorithm without replacement and with a caliper width of 0.2. Data are presented as the mean ± standard deviation, median (range) or number (percentage), as appropriate, for patient and surgical characteristics. Dichotomous data were analyzed using Fisher’s exact test, whereas continuous covariates were evaluated using the Mann–Whitney U test. All tests were two-sided and *P* values < 0.05 were indicated statistical significance. All statistical analyses were performed with JMP v.15.2 (SAS Institute, Cary, NC, USA; RRID: SCR_014242).

## Results

### Patient and surgical characteristics

A total of 86 patients with 88 lesions (I-D) and 52 patients with lesions (ICG-iv) were enrolled. Complete resection was achieved for all lesions. The characteristics of the patients and the clinical background of each group are summarized in Table 1. The mean age at surgery was 70.0 ± 9.5 years for the I-D group and 69.9 ± 8.8 years for the ICG-iv group; 57 patients (66.3%) in the I-D group and 30 patients (57.7%) in the ICG-iv group were male. A total of 59 patients (68.6%) in the I-D group and 35 (67.3%) in the ICG-iv group had a history of smoking. Among the I-D group and the ICG-iv group, the number of patients with restrictive pulmonary disease was 11 (12.8%) and 2 (3.9%) (**P* *= 0.131), and that with obstructive pulmonary disease was 32 (37.2%) and 11 (21.2%) (**P* *= 0.059). The median operative time was 164.5 min in the I-D group and 180 min in the ICG-iv group (**P* *= 0.123). There were no significant differences between the I-D and ICG-iv groups in perioperative outcomes, including prolonged air leakage, duration of drain placement, and postoperative hospital day. Regarding surgical characteristics, 88 lesions (I-D) and 52 lesions (ICG-iv) were included (Table 2). No significant differences in the consolidation diameter, consolidation tumor ratio, lobes with the lesion, or pathological classification of the lesions were observed. As to surgical approach, the ICG-iv group had significantly fewer cases of thoracotomy compared to the I-D group (*P* = 0.007). STAS was confirmed in 11 lesions (12.5%) in the I-D group and in 8 lesions (15.4%) in the ICG-iv group, and the difference was not significant (**P* *= 0*.*620). Complex segmentectomy was performed in 34 patients (38.6%) in the I-D group and 36 patients (69.2%) in the ICG-iv group, showing a significant difference (*P* < 0.001).

**Table 1:** Preoperative and perioperative patient characteristics (n = 138).

Characteristic	I-D group (n = 86)	ICG-iv group (n = 52)	*P* value
Age, years: mean±SD	70.0 ± 9.5	69.9 ± 8.8	0.895
Sex, male, n (%)	57 (66.3%)	30 (57.7%)	0.364
Smoking history, yes, n (%)	59 (68.6%)	35 (67.3%)	> 0.99
Restrictive pulmonary disease, yes, n (%)	11 (12.8%)	2 (3.9%)	0.131
Obstructive pulmonary disease, yes, n (%)	32 (37.2%)	11 (21.2%)	0.059
Surgical approach, n (%)			0.007
Thoracotomy	46 (53.5%)	16 (30.8%)	
Hybrid VATS	22 (25.6%)	18 (34.6%)	
Complete VATS	14 (16.3%)	18 (34.6%)	
RATS	4 (4.7%)	0 (0%)	
Operative time, min: median (range)	164.5 (104–334)	180 (91–282)	0.123
Blood loss volume, g: median (range)	5 (0–590)	2.5 (0–452)	0.295
Prolonged air leakage^†^, yes, n (%)	7 (8.1%)	7 (13.5%)	0.386
Duration of chest drainage, days: median (range)	2 (1-8)	2 (1-5)	0.526
Postoperative hospital stay, days: median (range)	8 (4-178)	7 (5-16)	0.659

^†^Grade three or higher complication according to the Common Terminology Criteria for Adverse Events version 5.0.

ICG-iv, indocyanine green intravenous administration; I-D, inflation-deflation; RATS, robot-assisted thoracoscopic surgery; SD, standard deviation; VATS, video-assisted thoracoscopic surgery.

**Table 2:** Surgical and pathological characteristics (n = 140).

Characteristic	I-D group (n = 88)	ICG-iv group (n = 52)	*P* value
Consolidation diameter, mm: median (range)	13 (1–47)	11 (0–27)	0.161
Consolidation/tumor ratio, %: median (range)	100 (16–100)	100 (0–100)	0.953
Localization, n (%)			0.533
Right upper lobe	19 (21.6%)	16 (30.8%)	
Right lower lobe	17 (19.3%)	11 (21.2%)	
Left upper lobe	34 (20.5%)	18 (34.6%)	
Left lower lobe	18 (20.5%)	7 (13.5%)	
Type of indication for segmentectomy			0.684
Positive indication	68 (77.3%)	38 (73.1%)	
Compromise indication	20 (22.7%)	14 (26.9%)	
Complex segmentectomy, n (%)	34 (38.6%)	36 (69.2%)	< 0.001
Pathological classification, n (%)			0.674
Primary lung cancer	70 (79.6%)	40 (76.9%)	
Metastatic lung tumor	17 (19.3%)	10 (19.2%)	
Others	1 (1.1%)	2 (3.9%)	
STAS positive, n (%)	11 (12.5%)	8 (15.4%)	0.620

ICG-iv, Indocyanine green intravenous administration; I-D, Inflation-deflation; STAS, Spread through air spaces.

### Evaluation of accuracy

**Fig 3:**
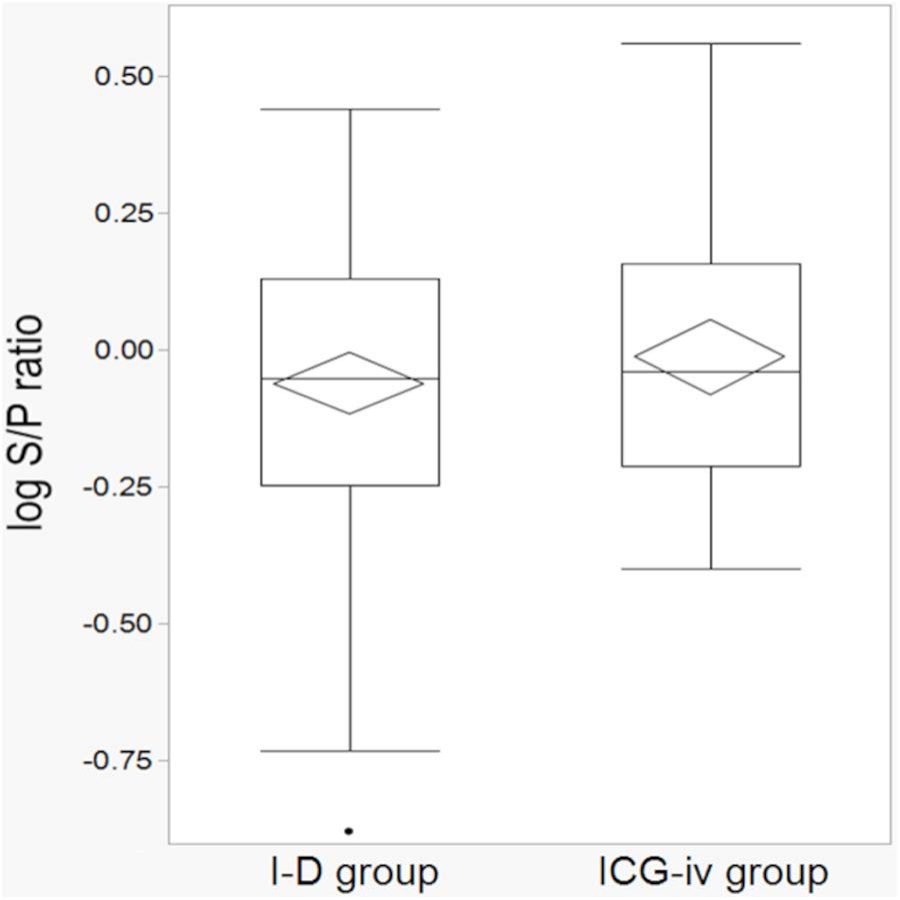
Logarithmic transformation of S/P ratio. The mean log S/P ratios were −0.061 (95% confidence interval: −0.118 to −0.004) in the I-D group and −0.013 (95% confidence interval: −0.082 to 0.056) in the ICG-iv group. ICG-iv, Indocyanine green intravenous administration; I-D, Inflation-deflation; S/P ratio, Surgical and planned margin ratio.

The list of items evaluated for accuracy is presented in Table 3. The median planned margin was 21.1 (4.2–88.1) mm in the I-D group and 17.5 (5.5–50.6) mm in the ICG-iv group, and the median surgical margin was 19 (5–77) mm in the I-D group and 18 (6–57) mm in the ICG-iv group, respectively. The median S/P ratio was 0.886 and 0.912 in the I-D and ICG-iv groups, respectively. The mean log S/P ratio was −0.061 (95% confidence interval: −0.118 to −0.004) in the I-D group and −0.013 (95% confidence interval: −0.082 to 0.056) in the ICG-iv group (*P* = 0.529) (Fig 3). Furthermore, the RMSLE values were 0.258 and 0.229 in the I-D and ICG-iv groups, respectively.

**Table 3:** Comparison of the accuracy of intersegmental plane identification using the Inflation-deflation method or indocyanine green intravenous administration method (n = 140).

Accuracy outcome	I-D group (n = 88)	ICG-iv group (n = 52)
Planned margin, mm: median (range)	21.1 (4.2–88.1)	17.5 (5.5–27)
Surgical margin, mm: median (range)	19 (5–77)	18 (6–57)
S/P ratio: median (range)	0.886 (0.132–2.750)	0.912 (0.398–3.636)
RMSLE	0.258	0.229

ICG-iv, Indocyanine green intravenous administration; I-D, Inflation-deflation; RMSLE, Root Mean Squared Logarithmic Error; S/P ratio, Surgical and planned margin ratio.

Following the propensity score matching procedure, 42 cases were selected from each group (S1 Table). The mean log S/P ratio was −0.100 (95% confidence interval: −0.181 to −0.020) in the I-D group and −0.010 (95% confidence interval: −0.089 to 0.070) in the ICG-iv group (*P* = 0.217) (S2 Table). The RMSLE values were 0.258 and 0.235 in the I-D and ICG-iv groups, respectively.

**Fig 4:**
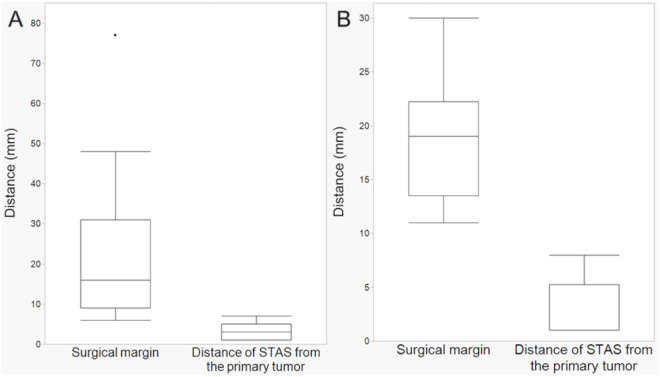
Relationships between the surgical margin and the distance of STAS from the primary tumor among the lesions with STAS. (A) I-D group (n = 11). The median surgical margin was 16 mm (6–77), and the median distance of the STAS from the primary tumor was 3 mm (1–7), showing a significant difference (*P* < 0.001). (B) ICG-iv group (n = 8). The median surgical margin was 19 mm (11–30), and the median distance of the STAS from the primary tumor was 1 mm (1–8), showing a significant difference (*P* < 0.001). ICG-iv, Indocyanine green intravenous administration; I-D, Inflation-deflation; STAS, Spread through air spaces.

Among the lesions with STAS, the median distance between the tumor rim and distal end of STAS was 3 mm (1–7) in the I-D group and 1 mm (1–8) in the ICG-iv group, which were significantly shorter than the surgical margin, both in the I-D group (*P* < 0.001) and in the ICG-iv group (*P* < 0.001) (Fig 4).

### Staining ability of intersegmental identification in the ICG-iv group

In the ICG-iv group, good staining of the intersegmental plane was observed in 44 patients with lesions (84.6%), and poor staining was observed in 8 patients with lesions (13.4%) (Table 4). Regarding the comparison of patient and surgical characteristics between the good and poor staining groups, the number of patients with obstructive pulmonary disease was 6 (13.6%) and 4 (50%), respectively, showing significant differences (**P* *= 0.035): there were no significant differences in the other factors, including sex, smoking history, restrictive pulmonary disease, segmentectomy in the lower lobe, and complex segmentectomy. A comparison of accuracy was made between the good and poor staining groups (S3 Table). The median S/P ratio was 0.886 and 0.954 in the good and poor staining groups, respectively. The mean log S/P ratio was −0.023 (95% confidence interval: −0.095 to −0.050) in the good staining group and 0.041 (95% confidence interval: −0.219 to 0.301) in the poor staining group (*P* = 0.676). The RMSLE values were 0.220 and 0.274 in the good and poor staining groups, respectively.

**Table 4:** Comparison of patient and surgical characteristics between good staining and poor staining group in the intersegmental plane with intravenous indocyanine green administration (n = 52).

Characteristic	Good staining group (n = 44)	Poor staining group (n = 8)	*P* value
Sex, male, n (%)	24 (54.6%)	6 (75%)	0.442
Smoking history, yes, n (%)	28 (63.6%)	7 (87.5%)	0.248
Restrictive pulmonary disease, yes, n (%)	5 (11.4%)	0 (0%)	> 0.99
Obstructive pulmonary disease, yes, n (%)	6 (13.6%)	4 (50%)	0.035
Segmentectomy in the lower lobe, n (%)	16 (36.4%)	2 (25%)	0.698
Complex segmentectomy, n (%)	30 (69.2%)	6 (75%)	> 0.99

## Discussion

We compared I-D and ICG-iv methods to determine the accuracy of intersegmental plane identification for patients who underwent lung segmentectomy. We found that the ICG-iv method could describe the intersegmental plane more accurately than the I-D method. The results of the subgroup analysis showed that patients with obstructive pulmonary disease were more likely to be in the poor staining group than in the good staining group in the ICG-iv method cohort.

The identification of the intersegmental plane by the ICG-iv method was reported to be of limited extent compared to that identified by the high-frequency jet ventilation system [5]. Two previous studies that evaluated the pathology of the dissection margin identified by the ICG-iv method showed high precision, using the planned margin identified by three-dimensional imaging from preoperative imaging studies as a control [6,7]. The present study compared the accuracy of I-D and ICG-iv methods in identifying intersegmental planes using pathologically measured surgical margins and planned margins calculated by three-dimensional imaging, which few previous reports have focused on. The findings of the present study could contribute to the existing literature and will contribute to the appropriate selection of a method of intersegmental identification for segmentectomies. Identifying intersegmental planes with high accuracy can allow safe margins to be secured, as well as the optimization of lung volume loss associated with pulmonary resection.

In the I-D method, attempts to infiltrate the target segment through a specific bronchus could result in difficulty in delineating the inflation-deflation line due to collateral ventilation pathways, such as the pores of Kohn, the canals of Lambert, and direct airway anastomosis [5,15]. Especially when the condition of the lungs is poor, such as in obstructive pulmonary disease, the identification of the intersegmental plane through the bronchus may be difficult. Once the lungs are inflated, it can take time to re-deflate, which may interfere with the surgical procedure. Limited visibility caused by inflated lung may affect the required size of the surgical incision. In the present study, a higher number of thoracotomy cases were observed in the I-D group. The ICG-iv method uses a different approach: intersegmental plane identification, derived from blood flow. This method may be less susceptible to the disadvantages mentioned above owing to pulmonary conditions; indeed, a high degree of accuracy was demonstrated in this study. However, in patients with obstructive pulmonary disease, emphysematous changes could occur with concomitant pulmonary hypertension, vascular remodeling, and reduction of the pulmonary vascular bed [5,16,17]. This may have influenced the visualization of the intersegmental plane using the ICG-iv method, albeit to a lesser extent than with the I-D method. Although a previous study has suggested that ICG use is generally applicable to pulmonary segmentectomy, including cases of obstructive pulmonary disease and complicated segmentectomy, it also reported that the time required for staining of the intersegmental plane to become clear after ICG intravenous administration could be shorter in patients with obstructive pulmonary disease, probably due to vascular abnormalities associated with emphysematous changes [5]. The reduced ICG imaging performance in patients with obstructive pulmonary disease in the present study may be associated with the shortened imaging time. In patients with such conditions, alternative identification techniques like the I-D method could be considered. If adequate delineation still cannot be achieved, anatomical identification of intersegmental veins may serve as an additional strategy. In cases where the tumor is located near the intersegmental plane and intraoperative tumor identification by palpation or visual inspection is expected to be challenging, identification methods such as CT-guided marking and bronchoscopic marking should be also considered.

The distance of the STAS from the primary tumor was significantly shorter than the surgical margin in both groups. Even if STAS was not along the same line from the primary tumor to the dissection plane, it is highly probable that complete resection of the lesion, including the STAS area, could have been achieved at the identified dissection line; however, this could not be determined because the central side of the dissection plane could not be evaluated in the pathological sample. It remains challenging to predict the presence and extent of STAS preoperatively, and its actual spread may exceed the extent suggested by imaging findings. Therefore, ensuring an adequate surgical margin is critical. In cases where STAS is confirmed, intensive postoperative follow-up may be warranted to monitor for potential recurrence. Ultimately, long-term follow-up is essential to assess local recurrence and verify the completeness of resection, particularly in relation to the STAS area.

The present study had some limitations. First, this was a single-institution retrospective study; and therefore, may have been subject to institutional bias. Although there was a possibility of selection bias in this way, propensity score matching was performed, to verify that there was a similar trend. Second, differences in patient background between the I-D and ICG-iv groups may have resulted from a case selection bias. Segmentectomy using the ICG-iv method was initiated in September 2021 at a single institution. Since then, either the ICG-iv or the I-D method has been used for segmentectomy at the discretion of the surgeon. Therefore, there were differences in the chronology of the cases between the two groups, which may have led to differences in patient characteristics. More lesions were resected by complex segmentectomy in the ICG-iv group: this surgical procedure must create multiple intersegmental planes and should increase the difficulty of securing surgical margins. To overcome this limitation, prospective randomized studies stemming from retrospective results are required. The third limitation is the validity of the pathological evaluation. As the resected lung was collapsed, despite the inflation of the lung by formalin solution injection through the resected bronchus for accurate margin measurement, the possibility that there was a difference from the original resection margin cannot be ruled out. The margin was defined by adding 5 mm as the thickness of the staple line during the pathological evaluation. However, in addition to the possibility that this may differ from the exact width of the staple, the reproducibility of the removal of the non-stapled portion of the resected lung when the staple line is removed may not be ensured. These factors could explain why the surgical margins were slightly shorter than the planned margins in both groups. In this study, we would like to emphasize that the evaluation methods would not differ from case to case because the same measurements were made in all cases and lesions.

In conclusion, this study demonstrated the superior accuracy of the ICG-iv method over the I-D method for lung segmentectomy. The ICG-iv method could be effective in lower lobe segmentectomy and complex segmentectomy, where intersegmental visualization is inherently more challenging. However, in patients with obstructive pulmonary disease, the visibility of intersegmental plane may be diminished when using the ICG-iv method alone. It is imperative to employ alternative identification techniques like the I-D method and anatomical identification of intersegmental veins, depending on the specific characteristics of each case. The efficacy of the ICG-iv method is expected to be further validated through the long-term local recurrence rate and/or prospective clinical studies.

## Supporting information

S1 Fig Intraoperative findings of the intersegmental plane description using the ICG-iv method. (A) Poor staining: the line delineated by intravenous ICG administration lacks clear linearity. (B) Good staining: the intended intersegmental plane is clearly visualized. ICG-iv, Indocyanine green intravenous administration.
(TIF)

S1 Table Patient characteristics prior to and following propensity score matching. ICG-iv, indocyanine green intravenous administration; I-D, inflation-deflation.
(DOCX)

S2 Table Comparison of the accuracy of intersegmental plane identification in the Matched Cohort. ICG-iv, Indocyanine green intravenous administration; I-D, Inflation-deflation; RMSLE, Root Mean Squared Logarithmic Error; S/P ratio, Surgical and planned margin ratio.
(DOCX)

S3 Table Comparison of the accuracy of intersegmental plane identification between good and poor staining group using the ICG-iv method. ICG-iv, Indocyanine green intravenous administration; RMSLE, Root Mean Squared Logarithmic Error; S/P ratio, Surgical and planned margin ratio.
(DOCX)
