# Supplementary figures and images for: Indocyanine green intravenous administration can more accurately identify the intersegmental plane than the inflation-deflation method in lung segmentectomy
Source: PLoS One. 2025 Aug 4;20(8):e0328362. doi: 10.1371/journal.pone.0328362 (PMC12321118; doi:10.1371/journal.pone.0328362)

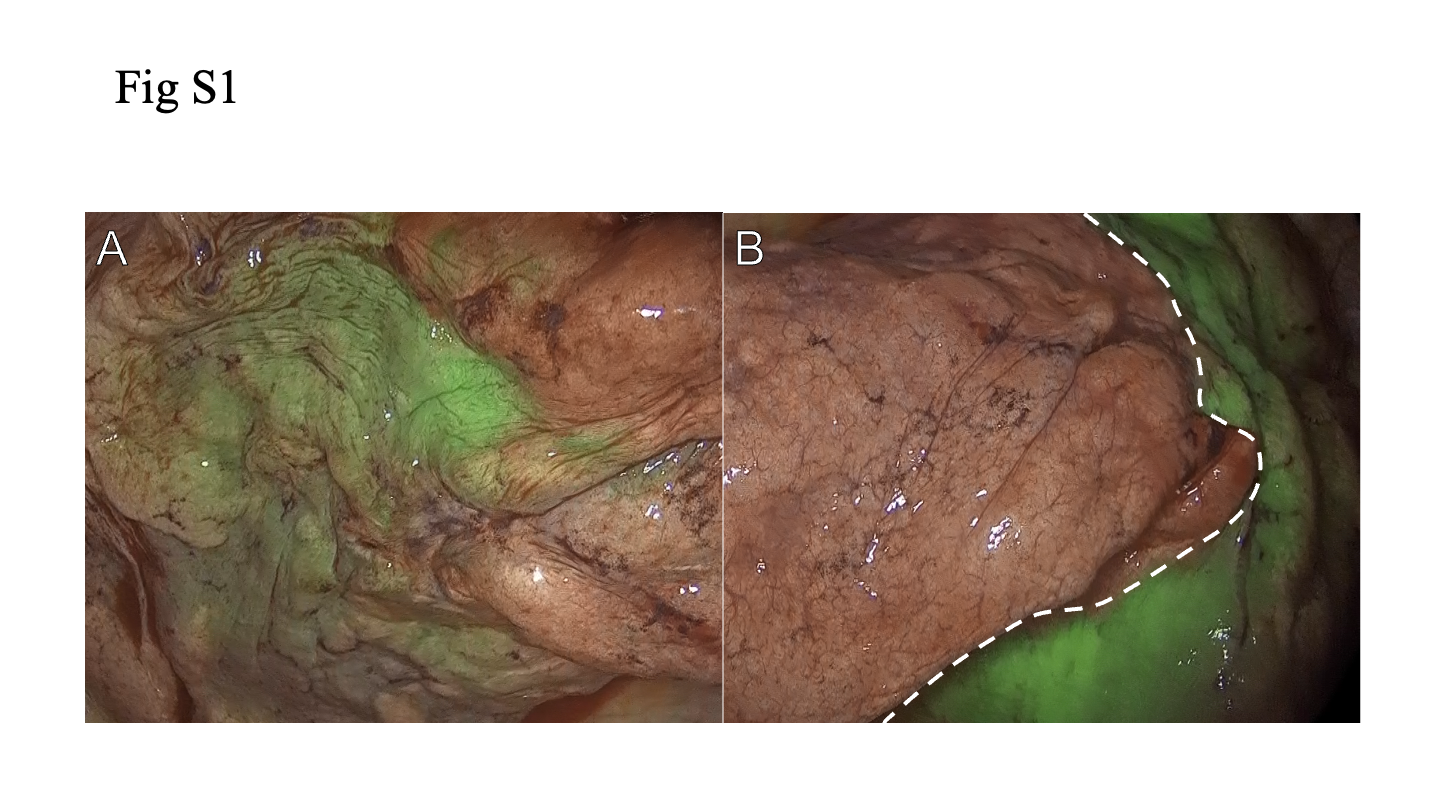

Supplement: S1 Fig — (A) Poor staining: the line delineated by intravenous ICG administration lacks clear linearity. (B) Good staining: the intended intersegmental plane is clearly visualized. ICG-iv, Indocyanine green intravenous administration. (TIF) [file pone.0328362.s001.tif]
