# Supplementary material for: Indocyanine green intravenous administration can more accurately identify the intersegmental plane than the inflation-deflation method in lung segmentectomy
Source: PLoS One. 2025 Aug 4;20(8):e0328362. doi: 10.1371/journal.pone.0328362 (PMC12321118; doi:10.1371/journal.pone.0328362)
Supplement: S1 Table — ICG-iv, indocyanine green intravenous administration; I-D, inflation-deflation. (DOCX) [file pone.0328362.s002.docx]

**Table S1.** **Patient characteristics prior to and following propensity score matching.**

| **Characteristic, n (%)** | **Unmatched group** | | | **Matched group** | | |
| --- | --- | --- | --- | --- | --- | --- |
|  | **I-D group**  **(n = 88)** | **ICG-iv group**  **(n = 52)** | ***P* value** | **I-D group**  **(n = 42)** | **ICG-iv group**  **(n = 42)** | ***P* value** |
| Sex, male | 58 (65.9%) | 30 (57.7%) | 0.368 | 24 (57.1%) | 25 (59.5%) | > 0.99 |
| Smoking history, yes, | 60 (68.2%) | 35 (67.3%) | > 0.99 | 27 (64.3%) | 26 (61.9%) | > 0.99 |
| Restrictive pulmonary disease, yes | 11 (12.8%) | 2 (3.9%) | 0.132 | 0 (0%) | 2 (4.8%) | 0.494 |
| Obstructive pulmonary disease, yes | 32 (36.4%) | 11 (21.2%) | 0.087 | 9 (21.4%) | 11 (26.2%) | 0.798 |
| Consolidation/tumor ratio, ≧ 50% | 69 (78.4%) | 40 (76.9%) | 0.836 | 31 (73.8%) | 32 (76.2%) | > 0.99 |
| Segmentectomy in the lower lobe, yes | 53 (60.2%) | 34 (65.4%) | 0.592 | 27 (64.3%) | 28 (66.7%) | > 0.99 |
| Complex segmentectomy, yes | 34 (38.6%) | 36 (69.2%) | < 0.001 | 23 (54.8%) | 26 (61.9%) | 0.658 |

ICG-iv, indocyanine green intravenous administration; I-D, inflation-deflation.
