# Supplementary material for: Indocyanine green intravenous administration can more accurately identify the intersegmental plane than the inflation-deflation method in lung segmentectomy
Source: PLoS One. 2025 Aug 4;20(8):e0328362. doi: 10.1371/journal.pone.0328362 (PMC12321118; doi:10.1371/journal.pone.0328362)
Supplement: S2 Table — ICG-iv, Indocyanine green intravenous administration; I-D, Inflation-deflation; RMSLE, Root Mean Squared Logarithmic Error; S/P ratio, Surgical and planned margin ratio. (DOCX) [file pone.0328362.s003.docx]

**Table S2. Comparison of the accuracy of intersegmental plane identification in the Matched Cohort.**

| **Accuracy outcome** | **I-D group**  **(n = 42)** | **ICG-iv group**  **(n = 42)** |
| --- | --- | --- |
| S/P ratio: median (range) | 0.778 (0.185–2.500) | 0.950 (0.398–3.636) |
| Log S/P ratio: mean (95% confidence interval) | -0.100  (-0.181 to -0.020) | -0.010  (-0.089 to 0.070) |
| RMSLE | 0.258 | 0.235 |

ICG-iv, Indocyanine green intravenous administration; I-D, Inflation-deflation; RMSLE, Root Mean Squared Logarithmic Error; S/P ratio, Surgical and planned margin ratio.
