# Supplementary material for: Indocyanine green intravenous administration can more accurately identify the intersegmental plane than the inflation-deflation method in lung segmentectomy
Source: PLoS One. 2025 Aug 4;20(8):e0328362. doi: 10.1371/journal.pone.0328362 (PMC12321118; doi:10.1371/journal.pone.0328362)
Supplement: S3 Table — ICG-iv, Indocyanine green intravenous administration; RMSLE, Root Mean Squared Logarithmic Error; S/P ratio, Surgical and planned margin ratio. (DOCX) [file pone.0328362.s004.docx]

**Table S3. Comparison of the accuracy of intersegmental plane identification between good and poor staining group using the ICG-iv method.**

| **Accuracy outcome** | **Good staining group**  **(n = 44)** | **Poor staining group**  **(n = 8)** |
| --- | --- | --- |
| S/P ratio: median (range) | 0.886 (0.398–3.636) | 0.954 (0.537–3.382) |
| Log S/P ratio: mean (95% confidence interval) | -0.023  (-0.095 to -0.050) | 0.041  (-0.219 to 0.301) |
| RMSLE | 0.220 | 0.274 |

ICG-iv, Indocyanine green intravenous administration; RMSLE, Root Mean Squared Logarithmic Error; S/P ratio, Surgical and planned margin ratio.
